# Advancing malaria reactive case detection in a Zambia-like setting: A modeling study

**DOI:** 10.1371/journal.pgph.0004288

**Published:** 2025-02-20

**Authors:** Chilochibi Chiziba, Japhet Chiwaula, Busiku Hamainza, Sheetal Silal

**Affiliations:** 1 Modelling and Simulation Hub, Africa, Department of Statistical Sciences, University of Cape Town, Cape Town, South Africa; 2 Ministry of Health, National Malaria Control Centre, Lusaka, Zambia; 3 Centre for Global Health, Nuffield Department of Medicine, Oxford University, Oxford, United Kingdom; Aarhus University: Aarhus Universitet, DENMARK

## Abstract

In Zambia-like settings, asymptomatic and clinical carriers not seeking treatment further complicate malaria elimination, making reactive case detection (RCD) essential for identifying undetected infections. However, RCD faces operational hurdles, including resource shortages, logistical challenges, limited community health workers (CHWs), and limitations in availability and sensitive rapid diagnostic tests (RDTs). Prioritizing specific improvement measures is critical to enhance intervention outcomes. A mathematical model of malaria transmission for low-transmission areas (fewer than 200 cases per 1,000 annually) was developed using published data to simulate RCD. This model assessed the impact of potential improvement measures designed to address the identified operational challenges affecting RCD. Improvement measures included increasing CHWs, adjusting response times, improving RDT sensitivity, and incorporating focal mass drug administration (fMDA). A shortage of CHWs and limited availability of RDTs have the most negative impact on RCD’s ability to reduce cases. In scenarios where CHWs or RDT availability for RCD were reduced by 50%, annual cases increased by approximately 22%. Only the incorporation of fMDA as an improvement measure succeeded countering the situation, resulting in a 43% reduction. Increasing CHWs to offset RCD inefficiencies caused by limited RDT sensitivity and difficulties finding individuals reduced cases by approximately 13 and 14%, respectively, reducing more cases than improving reaction time or increasing the screening radius. Although RCD is prone to challenges, the manipulation of improvement measures such as CHWs and fMDA provides promise for RCD to contribute towards malaria elimination. However, the participation of CHWs is voluntary and primarily motivated by informal incentives, often provided by donors. Finding sustainable means to ensure the sufficient availability of CHWs may guarantee continued RCD contributions toward maintaining stable malaria prevalence. More research is required to explore the application of RCD in archetypical transmission areas suitable for RCD as improvement measures to the identified challenges hindering RCD.

## Author summary

This study used mathematical modeling to investigate the challenges impacting reactive case detection (RCD), an approach that involves testing individuals living near a confirmed malaria case in low-transmission areas of Zambia. Furthermore, we explored the impact of potential improvement measures designed to address these identified challenges. Shortages of community health workers (CHWs) and rapid diagnostic tests (RDTs) were identified as the biggest obstacles, with a half reduction in the number of CHWs or RDTs leading to an increase in annual malaria cases by about 22%. Treating everyone near a known malaria case was the only measure that effectively countered these challenges. Increasing CHWs to address RCD inefficiencies caused by limited RDT sensitivity or difficulties in locating individuals reduced more malaria cases than improving response time or expanding the screening radius. We conclude that while RCD faces many challenges, measures such as increasing CHWs or treating everyone near a known malaria case show promise for enhancing RCD’s contribution to malaria elimination. However, as CHWs often work voluntarily and rely on donor incentives, sustainable solutions are needed to ensure their continued availability. Further research is required to determine how RCD can be most effectively applied and improved across different settings.

## Introduction

### Malaria trends at health facility catchment area level in Zambia

Malaria has proved difficult to eradicate despite being preventable and treatable, likely due to its complex transmission dynamics [1]. Although some countries have successfully eliminated malaria, countries in sub-Saharan Africa still carry the highest burden, accounting for approximately 90% of the 263 million cases reported globally [2]. Furthermore, the malaria burden varies significantly across sub-Saharan Africa and within countries, including Zambia [2,3]. Complicating the implementation of intervention strategies are the complexities of transmission and heterogeneity as well as the presence of the most efficient vector, individuals who fail to seek treatment despite exhibiting symptoms and those who remain asymptomatic yet capable of transmitting the disease [4,5].

Although Zambia is not among the top contributors to global malaria cases, as of 2021, 57% of its population lived in regions classified as having low to very low malaria risk (less than 200 cases per 1,000 people per year) [3]. However, malaria risk exhibits spatial heterogeneity across smaller spatial boundaries, such as health facility catchment areas (HFCAs) [3,6]. From 2017 to 2021, the distribution of cases per 1000 people per year at HFCA level remained relatively stable, except for a notable increase in percent of HFCAs that are classified as high risk (> 500 cases per 1000 per year) from 24% in 2019 to 47% in 2020 [3]. As Zambia works to increase the number of low malaria-risk HFCAs through various interventions, the potential for a resurgence of cases in these regions remains high [5]. This is because the proportion of asymptomatic individuals with low parasite densities among the infected population rises, as malaria transmission rates decrease [5]. Despite being less infectious than symptomatic cases, these individuals form an asymptomatic reservoir capable of transmitting parasites in areas where vectors are present [5]. Furthermore, the non-detection of asymptomatic individuals is influenced by the limited availability of cost-effective diagnostic tools that are sensitive enough to detect low levels of parasitaemia [7]. To combat the resurgence of cases, interventions including reactive case detection (RCD) are strategically implemented in low transmission areas to target asymptomatic infections and symptomatic individuals not seeking treatment, offering treatment to halt transmission without the need for universal testing or treatment as part of the case management strategy [3,5,8]. Aside from RCD, other interventions, such as mass drug administration (MDA), are implemented to clear parasites in specific areas [3]. Additionally, other forms of MDA, such as focal mass drug administration (fMDA), are conducted to target smaller sites with a known positive case(s) [3]. Both RCD and fMDA are triggered by the identification of a positive case; however, RCD’s incorporation of testing ensures that individuals receiving treatment are confirmed to have parasites [3].

### Community health workers and the Zambian healthcare system

The operational context of malaria case management in Zambia involves a multi-tiered healthcare system, with interventions implemented at hospitals, health centres, and health posts [3]. All health facilities serve as diagnostic and treatment centres for malaria, with hospitalization for severe cases limited to facilities with admission capacity [3]. Suspected malaria cases at these facilities are subjected to parasitological testing (using RDTs or microscopy) [3]. Additionally, malaria programs are integrated within the healthcare system at all levels, from national to community [3]. Community Health Workers (CHWs) and Community Health Assistants (CHAs) play crucial roles in diagnostics and treatment at the community level through interventions such as RCD [3]. Both CHWs and CHAs are expected to manage cases at the community level using essential supplies such as RDTs and ACTs [3]. However, CHWs typically serve voluntarily and are often involved in a range of facility-based activities beyond malaria, making it challenging for them to fully dedicate their efforts to malaria-specific interventions like RCD [3,9]. Despite these constraints, CHWs are expected to support HFCAs with fewer than 200 annual malaria cases in conducting RCD follow-ups [3].

### Previous studies on reactive case detection in Zambia

In addition to the National Malaria Elimination Centre recommended RCD approach. Various research focused RCD variants and evaluations have previously been conducted. These studies indicates that RCD is effective in several ways but also impeded by various challenges. A mathematical modelling study conducted by Gerardin et al. (2017) used household locations, demographics, and malaria prevalence data to train an agent-based model to assess the effectiveness of RCD based on different transmission profiles, which included, “low-transmission, high household density; high-transmission, low household density; and high-transmission, high household density [9].” The simulation findings estimated that RCD is only effective in areas that have newly become low transmission areas [9]. Also, Chitnis et al. (2019), in a theoretical modelling paper that used Zambian data found that it is more important to increase the number of index cases followed than to increase the number of neighbours tested per index case, if RCD is to be effective [10]. Similarly, Reiker et al.’s (2019) mathematical modelling study suggests that RCD is ideal in areas where transmission is initially low, and that increasing radius yields relatively better case detection [11]. Furthermore, Larsen et al. (2017) and Bhondoekhan et al. (2020) suggest that prioritizing locations with high environmental susceptibility to malaria transmission during RCD operations is crucial in detecting cases in low transmission areas [4,12]. Additionally, all studies on RCD in Zambia agree that RCD’s efficacy can be improved and that, on its own, it may not lead to malaria elimination in low transmission areas. However, if complemented with vector control and preventative chemotherapy interventions, it may realistically lead to elimination [4,9–14]. Most importantly [10,11], conclude that prevalence reduction due to RCD is mainly determined by the proportion of all infections identified within a specific timeframe [10,11].

### Informing operational decision-making for reactive case detection

While the studies on RCD in Zambia provide valuable insights, recent advancements in malaria interventions will affect RCD outcomes differently, such as advancements in malaria rapid diagnostic test (RDT) sensitivity. Some of these studies compare the circumstances/settings in which RCD is most efficient. However, their applicability for informing operational decision-making may be limited, considering the operational challenges that impede RCD implementation in resource-constrained settings. These challenges frequently result in relatively fewer detections by RCD, further reducing its effectiveness [5]. An evaluation conducted by Searle et al. (2016) in the low-transmission regions of the Southern Province of Zambia highlighted several operational hurdles hindering the implementation of RCD. These hurdles included inadequate supplies of RDTs, a shortage of community health workers (CHWs), logistical complexities, difficulties in reaching residents in designated households, and the limited sensitivity of RDTs [5]. These challenges directly affect the ability of RCD to identify undetected infections [5]. Given the competing priorities faced by those implementing malaria interventions like RCD, understanding which improvement measures to prioritize based on specific circumstances can help address deficiencies in intervention outcomes. It is important to note that the operational challenges highlighted can be generalized to other countries with similar settings and RCD setup, hence the Zambia-like reference. However, these challenges may differ across HFCAs within a “Zambia-like” context. For instance, the availability of CHWs may vary based on factors such as regional differences, workload, donor presence, and resource allocation, while the availability of supplies like RDTs may depend on the level of accessibility [2,3].

This study takes a distinctive approach by addressing the practical challenges faced by implementers, rather than evaluating interventions under idealized conditions, which are rarely encountered in real-world scenarios. Zambia’s National Malaria Elimination Centre and its partners have demonstrated a strong commitment to adopting innovative strategies for malaria elimination, including HFCA-level risk stratification to prioritize interventions and the integration of mathematical modeling [3]. Building on the existing microstratification strategy, which focuses on HFCA-level interventions, this study aims to further identify optimal solutions to the challenges of implementing RCD at the HFCA level, leveraging routine data such as that from the Malaria Indicator Survey (MIS), and publications.

Overall, the purpose of this study was to use mathematical modelling to investigate the impact of various literature-informed challenges affecting RCD in reducing malaria cases and to assess the effect of different potential countermeasures in addressing these challenges. The countermeasures include increasing the CHW workforce, improving reaction time, changing diagnostics tests, or pivoting fMDA. These measures are targeted for the most common situations that lead to inefficient implementation of RCD in low transmission areas in Zambia to relatively better inform operational decisions.

## Methods

### Study site

**Fig 1:**
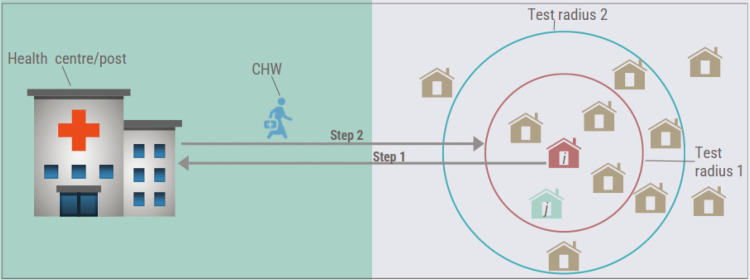
Schematic describing the operation of RCD at the health centre or health post in Zambia’s low transmission areas. In the schematic, *i* represents the index case’s household, while household *j* represents the household with a positive case not visiting a health facility.

In this study, we simulate a single hypothetical low-transmission health catchment area. Specifically, the study used public data from randomized control trials and cross-sectional studies conducted in low transmission areas of the Southern Province in the years 2014 to 2018 [4,5,9,11,12,14–16]. In Zambia, RCD is implemented in low transmission areas as stratified by HFCA [3]. In such areas, a positive malaria case at the health facility or post triggers an RCD investigation, which is carried out by CHWs assigned to the health post near the index case, as visualized by the schematic in Fig 1 [3,15]. In the figure, a positive malaria index case *i* (from the red housing unit) visits the health facility for any reason, such as after exhibiting symptoms. Once the health facility establishes that the individual is positive for malaria, a CHW is assigned to visit the location where the index case resides (Fig 1). Once at the location, the CHW tests individuals within the program-defined test radius with the potential of capturing other malaria-positive individuals such as *j* (blue housing unit) who did not seek treatment (Fig 1). In the RCD schematic, two test radius examples have been illustrated to show the potential to increase the radius to capture more households (Fig 1). However, Zambia’s recommended radius is 140 meters. Furthermore, one HFCA serves approximately 10,000 people, and a health post serves about 500 to 1000 people [4,5]. However, challenges such as shortage of CHWs outlined in the introduction may occasionally outer the dynamics of RCD implementation. Nevertheless, this study assumes a perfect RCD as the baseline scenario, which is further described later in the paper.

### Malaria reactive case detection model

**Fig 2:**
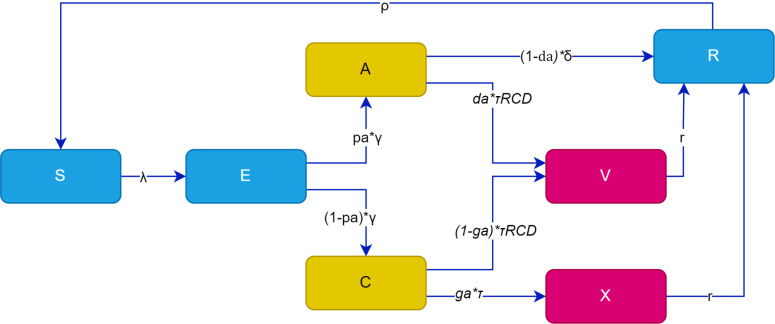
RCD model flow diagram with compartments S (Susceptible), E (Exposed), A (asymptomatic), C (Symptomatic/clinical), V (treatment through RCD), X (treatment at health facility), and R (recovered). The description of parameters governing movements through the compartments are described in Table 1.

To mimic the operation of RCD at the HFCA level, a deterministic non-linear ordinary differential equation (ODE) model was developed to simulate malaria transmission and RCD implementation visualized in Fig 2. The figure provides a simple overview of the RCD malaria transmission model, which takes a common susceptible‐exposed‐infected‐recovered (SEIR) model format with added treatment compartments. The SEIR model was chosen for its simplicity and compatibility with the available data. The model includes the human population only. In this model (Fig 2), individuals progress through distinct compartments representing various stages of infection and treatment. Initially, individuals are categorized as susceptible (S), signifying their vulnerability to malaria acquisition. Following exposure to the malaria parasite, individuals transition to the exposed (E) compartment, indicative of infection without immediate infectiousness. Subsequently, individuals may progress to either the asymptomatic (A) or symptomatic (C) compartments, contingent on the manifestation of malaria symptoms. The symptomatic individuals may undergo therapeutic intervention at a health facility, leading them to the treatment (X) or treatment through RCD (V) compartments, where treatment is administered. Furthermore, some of the asymptomatic (A) individuals may also transition into the treatment through RCD compartment V or recover naturally. Ultimately, individuals in the treatment compartments V and X and asymptomatic individuals not treated through RCD advance to the recovered (R) compartment, reflecting either clearance of the infection or the establishment of partial immunity. Table 1 summarize the parameter definitions that govern the transitions between compartments depicted in Fig 2. In this table, the treatment-seeking rate and the proportion of clinical infections treated at health facilities are proxied by the “percentage of children ages 0–59 months with fever reporting a finger/heel stick” and “percentage of children aged 0–59 months with fever who received artemisinin-based combination therapy (ACT)”, respectively. Furthermore, the parameter values were obtained from studies that specifically estimated parameter values from Zambia and other countries tailored to the dynamics of low transmission areas. The source studies are referenced in Table 1.

**Table 1:** Model parameters, values, descriptions, and sources.

Symbol	Definition	Value	Range	Source
**a**	Human feeding rate per mosquito (per day)	0.33	(0.10, 1)	[20]
**b**	Transmission efficiency from mosquito to human (per day)	0.022	(0.010,0.27)	[20]
**c**	Transmission efficiency human to mosquito (per day)	0.24	(0.072, 0.64)	[20]
**pa**	Proportion of asymptomatic infections	0.395	(0.2,0.49)	[15]
**da**	Proportion of asymptomatic infections that get screened and treated through RCD	0.22	(0.15,0.3)	[5]
**ga**	Proportion of clinical infections that get treatment at health facility	0.97	(0.84,0.99)	[6]
**γ**	Rate of onset of infectiousness in humans (Incubation rate; per day)	0.071	(0.06,0.08)	[19,21]
**τRCD**	Rate at which infectious population is screened and treated via RCD (per day)	Estimated during analysis		
**λ**	Force of infection	Estimated during analysis		
***τ***	Treatment seeking rate (per day)	0.59	(0.52,0.66)	[6]
**δ**	Natural recovery rare (per day)	0.0035	(0.0014, 0.017)	[19,22]
**r**	Recovery rate after antimalarial treatment (AL; per day)	0.167	(0.125, 0.25)	[19,23]
**ρ**	Loss of immunity (per day)	0.0027	(0.000055,0.011)	[20]
**θ**	Relative infectiousness of asymptomatic infections	0.467	(0,0.50)	[24,25]
**ζ**	Relative infectiousness of treated infections	0.04	(0.0,0.25)	[25]
**γ**_**m**_	Rate of onset of infectiousness in mosquitoes (per day)	0.1	(0.07, 0.2)	[20,25]
**μ**_**m**_	Mosquito mortality rate (per day)	0.033	(0.0010,0.10)	[20]
**m**	mosquito-human ratio	0.39	(0.34,0.46)	[26]
**φ**	Month of peak transmission per month	1	(0,1)	CHIRPS data
**α**	Amplitude of seasonal variation	0.5	(0,1)	Estimated during analysis

In this study, we assume that mosquitoes have a relatively rapid generation turnover and are highly responsive to changes in the proportion of infected humans [17–19]. Therefore, mosquito population can be considered to be at equilibrium with respect to changes in the human population [17–19]. Hence, we simplify the vector equations and determine the number of humans who become infected under the prevailing model conditions by focusing on a single force of infection. That way, it allows us to run our simulation without considering the changes in vector dynamics but changes in the force of infection. We derived the force of infection and model equations in the supplementary file S1 Text. Furthermore, we forced a seasonality equation (also described in the supplementary file) to the force of infection to mimic Zambia’s seasonal transmission pattern using rain data from the Climate Hazards Group InfraRed Precipitation with Station (CHIRPS) data. The final force of infection (λ) is summarized in Equation 1, with its parameters detailed in Table 1.


$$\text{λ}=\left(sea\left(t\right)\right)\frac{a^{2}bcm\frac{I}{P}}{\left({\text{µ}}_{m}+ac\frac{I}{P}\right)}\left(\frac{{\text{γ}}_{m}}{{\text{γ}}_{m}+{\text{µ}}_{m}}\right)$$ (1)

Where seasonality over time *(t); sea(t)* is summarized in equation 2.


$$sea\left(t\right)=1+rain*\alpha *\text{cos}(2\pi \left(t-\varphi \right)$$ (2)

### Reactive case detection rate

We explored RCD rate equations from previous work by Njau and Silal et al. (2021) and Das et al. (2022) to ascertain the effect of RCD in our model. Both equations produced similar results; however, we settled on the approach by Njau and Silal et al. (2021) due to its simplicity [25,27]. The rate of detecting cases, RCD τRCD, is defined in Equation 3, based on the work of Njau and Silal et al. (2021), where $cov_{RCD}$ is the proportion of index cases that are followed up, incidence is the number of new index cases at the health centre, while sample is the number of people screened that are within the proximity of the index case [25]. Furthermore, the pop and RDTsensitivity are population in the model and the sensitivity of RDTs used during the intervention, respectively [25]. Additionally, the value 1.5 in the equation is a household inflation factor, which increases the number of cases detected at the household level to account for the random testing assumption. The 0.5 (50%) inflation, drawn and established from previous work by Njau and Silal et al. (2021), is used to adjust for the relative likelihood of detecting cases in households in the RCD sample compared to random testing within the model. In our study, this inflation factor is applied because the model does not account for the spatial distribution of households, which can influence the effectiveness of RCD activities [25].


$$\text{τRCD}=\left(cov_{RCD}*incidence\left(1+\frac{1.5sample}{pop}\right)\right)\text{*RDTsensitivity }$$ (3)

To incorporate CHWs, we define $cov_{RCD}$ as a function of CHWs and index cases as presented in equation 4, where we estimated the numerator as the average number of index cases investigated by a single CHW per day based on the information provided by Larsen et al. (2017) [4]. Here, 333 CHWs investigated about 854 index cases in one year in some low transmission areas of Zambia. Therefore, we divided the total number of index cases investigated by the number of CHWs and then by the number of days in a year, which gives us equation 4. We assume that CHWs work every day to make the modelling process less complicated and to account for some intensified RCD days.


$$cov_{RCD}=\frac{0.007CHWs}{Indexcases}$$ (4)

We incorporated radius in the model by making sample a function of estimated average population of 0.56 to 1.3 screened per square meter based on RCD screenings done from [5]. Similarly, to incorporate the number of available residents screened, we reduced sample by the respective scenario required percentage.

### Incorporating reactive Focal Mass Drug Administration

We additionally explored reactive fMDA as one of the measures to reduce malaria cases by interrupting transmission in the HFCA. Thus, all individuals within the proximity of the index case receives treatment, implying that, infectioned individuals with low levels of parasitaemia who would not have been detected by RDT gets cleared of parasites [16,22,24,28]. Similarly, the susceptive and exposed are prevented from transitioning to the infectious category to transmit, thereby interrupting the transmission cycle with the HFCA [16,22,24,28]. Mathematical equations describing the incorporation are described by supplementary file S1 Text equation 21.

### Reactive case detection improvement scenarios

Various scenarios were formulated to address common situations that often result in inefficient implementation of RCD, namely, “Baseline”, “Increased number of CHWs”, “Increased Radius (250 meters)”, “Increased radius (450 meters)”, “Increased radius 250 + 50% more CHWs”, “Increased radius 450 + 50% more CHWs”, “Improved reaction time” (IRT) and “Increased RDT Sensitivity”. The purpose of formulating these scenarios was to assess their potential to achieve results similar to or better than the baseline. In these simulations, we assume that other interventions implemented to keep the low transmission status in the HFCA remain consistent and that situations only affected RCD implementation.

The baseline configuration assumed 20 community health workers per health centre dedicated to RCD, with each CHW representing a health post serving 500 individuals. This configuration was based on the estimated population of 10,000 in the HFCA, with an area coverage radius of 140 meters per index case [4,5,15]. The reaction time, which refers to the time taken to respond to reported cases, was set at three days. No fMDA was implemented, and RDTs had a sensitivity of 84%.

In the “Increased number of CHWs” scenario, the number of CHWs per HFCA was increased to 30, while all other parameters remained unchanged from the baseline scenario. This adjustment aimed to improve coverage and response capabilities within the same coverage radius. In the “Increased Radius (250 meters)” scenario, the coverage area was expanded by increasing the radius to 250 meters, while keeping the number of community health workers and other parameters constant. Similarly, in the “Increased radius (450 meters)” scenario, the coverage radius was further increased to 450 meters.

Furthermore, we explored scenarios that combined an increase in the number of CHWs (30) with an increase in the coverage radius, both for 250 and 450 meters. These scenarios aimed to improve both personnel and coverage area to improve intervention outcomes. Also, we simulated fMDA with the assumption that acceptance, coverage and drug efficacy all remain constant. The scenarios “Improved reaction time” (IRT) and “Increased RDT Sensitivity” had the same parameters as the baseline, except for changes in the reaction time (2 days) and RDT sensitivity (99%). The RDT sensitivity was set at this level, with the potential to be replaced by polymerase chain reaction as the testing option.

### Challenges/situations affecting reactive case detection and potential improvement scenarios

Table 2 summarizes the common situations mentioned elsewhere that may lead to reduced efficacy of implementation of RCD. It also presents simulated improvement scenarios to assess their potential for maintaining or improving the effectiveness of RCD when faced with potential impediments. Here, we assumed that if the health facility is faced with a situation such as a shortage of CHWs, it is unable to immediately replenish them but requires conducting a different improvement measure that may maintain or improve the results of RCD.

**Table 2:** Situations and potential improvement scenarios.

Situation	Improvement scenarios simulated
**Shortage of community health workers (50% less CHWs)**	Improved reaction time (from three to two days)
	Increased RDT sensitivity (from 84 to 99%)
	Incorporating fMDA (Replaced RCD)
**Limited availability of RDTs (50% less RDTs)**	Incorporating fMDA (Replaced RCD)
	Increased reaction time (from three to two days)
**Limited sensitivity of RDTs (70% sensitivity)**	Increased radius 250m (from 140 to 250m)
	Increased radius 450m (from 140 to 450m)
	Increased reaction time (from three to two days)
	Increased number of CHWs (50% more CHWs)
	Increased radius 250 ^+^ 50% more CHWs
	Increased radius 450 ^+^ 50% more CHWs
**Difficulties in finding residents in designated households (50% availability)**	Improved reaction time
	Increased RDT sensitivity
	Increased number of CHWs (50% more CHWs)

### Malaria risk stratification

In Zambia, there is an annual program that stratifies each HFCA based on malaria transmission intensity levels. These levels are categorized as “no malaria” (level 0), “very low” (level 1, between 0 and 50 cases per 1000 population/year), “low” (level 2, between 50 and 200 cases per 1000 population/year), “moderate” (level 3, between 200 and 500 cases per 1000 population/year), and “high” (level 4, with over 500 cases per 1000 population/year [3,29]. In this study, we used the same stratification as thresholds to ascertain that our model outputs are within the malaria risk classifications and to inform the impact of RCD hurdles and their respective improvement measures while assuming that all other interventions remain implemented at a constant rate. However, our model was not calibrated to any specific HFCA. Our model was run at a HFCA level (10,000 individuals) and day as the unit of change. Therefore, for the HFCA to qualify as a low transmission area (less 200 cases per year), it is required to have approximately less than 5.48 cases per day, thus, a sum of 2000 cases per 365 days.

### Uncertainty intervals for model results

To account for model uncertainty, we generated uncertainty intervals for all the results. We did this by running 100 simulations using randomly generated parameter values within the lower and upper bounds for all parameters in Table 1 in each model scenario. After generating results from 100 simulations for each scenario, we grouped daily malaria case values and obtained the median, 5th, and 95th quantile values for each day. The median value was used as the central value for each day, while the 5th and 95th quantile values were used as the lower and upper uncertainty values, respectively. This process was repeated for all model scenarios.

### Model validation and sensitivity analysis

Before running scenarios, we conducted several model validations by running several model simulations to confirm the relationships between the parameters and the output of interest (malaria cases). The validation checks focused on examining changes in the cumulative malaria cases when each parameter was altered. Additionally, we verified the direction of the relationship between the parameters and the cumulative cases to ensure that the model parameters aligned with expectations from the literature. Lastly, we performed a multivariable regression using standardized random parameter values within the lower and upper bounds for each parameter, with the model output of interest (cumulative malaria cases) as the dependent variable. The regression results were used to determine the statistical relationships between the parameters and the model output to establish parameter sensitivity. Results for these analyses are presented as supplementary files in S1 Fig, S2 Fig, and S2 Table.

## Results

### Impact predictions for key challenges affecting the efficiency of reactive case detection

**Fig 3:**
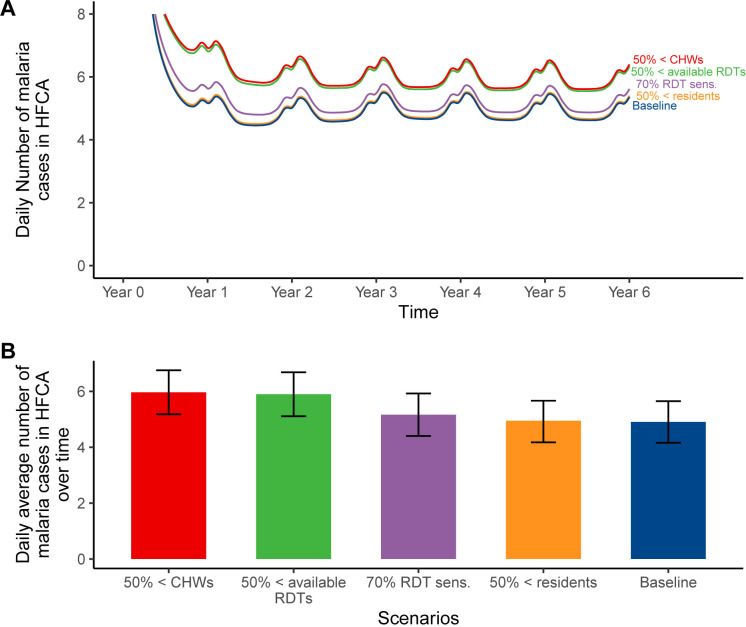
Impact prediction of key challenges impacting the efficiency of RCD: (A) daily incidence in the HFCA; (B) average number of malaria cases over six years by scenario with their respective average uncertainty intervals. In this simulation, the four scenarios are compared to the baseline and ability to influence malaria risk stratification status. Scenarios “50% <available RDTs” indicates a 50% reduction in the availability of RDTs, “50% <CHWs” represents a 50% shortage of CHWs, “50% <residents” denotes that only 50% of residents are available in designated households, and “70% RDT sens.” represents the use of RDTs with 70% sensitivity. Baseline scenario had 20 CHWs, 140 radius, 3 days reaction time, no fMDA, and 0.84 RDT sensitivity.

Fig 3A shows daily malaria cases for a hypothetical HFCA with seasonality affecting the annual trend while Fig 3B shows the average daily cases over the six years period by scenario. In the figure (Fig 3A and B), the baseline (*blue*) represents a scenario in which all interventions including RCD are being implemented in accordance with recommended guidelines for low transmission areas. In the figure, a 50% reduction in the number of CHWs (red) and the availability of RDTs (green) for RCD resulted in the highest deviation of malaria cases from the baseline (Fig 3) compared to the use of less sensitive RDTs (*purple)* and not finding 50% of individuals in their households (*orange*), which had the least impact (Fig 3). In contrast to the baseline scenario with 179 annual cases per 1,000 population (4.90 daily cases per HFCA), a scenario with a 50% reduction in CHWs (orange) resulted in approximately 218 annual cases per 1,000 population (5.97 daily cases per HFCA), while a 50% reduction in available RDTs (red) led to 215 annual cases per 1,000 population (5.90 daily cases per HFCA), as shown in Fig 5. Furthermore, in a scenario with 70% sensitive RDTs (green) cases increased to approximately 189 annual cases per 1,000 population (5.16 daily cases per HFCA). Failing to locate 50% of residents (purple) resulted in 180 annual cases per 1,000 population (4.95 daily cases per HFCA), as also shown in Fig 5.

### Impact predictions of RCD improvement measures for shortage of community health workers

**Fig 4:**
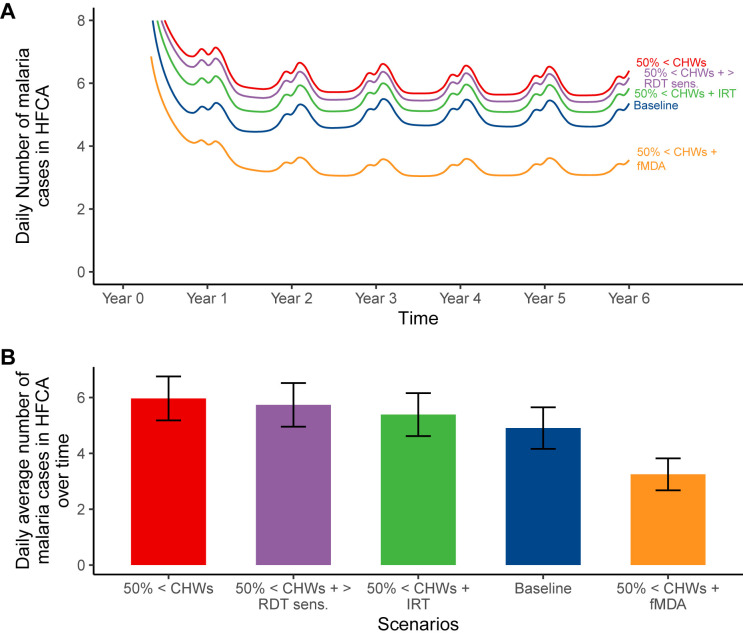
Impact predictions of improvement measures for shortage of CHWs: daily incidence in the HFCA; (B) average number of malaria cases over six years by scenario with their respective average uncertainty intervals. In this simulation, the number of CHWs was reduced by 50%, represented by “50% <CHWs,” i.e., from 20 to 10 CHWs per 10,000 population. The “50% <CHWs + RDT sens.” scenario depicts the use of more sensitive (99%) RDTs as an improvement measure to counter the impact of reduced CHWs. Similarly, “50% <CHWs + fMDA” and “50% <CHWs + IRT” represent using fMDA and improving the reaction time from three to two days, respectively as countermeasures. Baseline scenario had 20 CHWs, 140 radius, 3 days reaction time, no fMDA, and 0.84 RDT sensitivity.

In the scenario of a 50% shortage of CHWs; Fig 4 demonstrate that incorporating fMDA (*orange)* as an improvement measure results in relatively fewer cases of 119 annual cases per 1000 population (3.25 daily cases per HFCA) compared to all other countermeasures, including the baseline (*blue) with* 179 annual cases per 1000 population (4.90 daily cases per HFCA). Increasing RDT sensitivity purple), even up to 99%, as an improvement measure made the least difference with 209 annual cases per 1000 population (5.74 daily cases per HFCA) compared to the 50% CHW shortage scenario with approximately 219 annual cases per 1,000 population (5.97 daily cases per HFCA) as shown in Fig 4. However, improving the reaction time (*green)* from the recommended three days to two days resulted in relatively fewer cases of approximately 196 annual cases per 1000 population (5.39 daily cases per HFCA), but not enough to maintain or achieve fewer cases than the baseline (Fig 4).

### Impact predictions of RCD improvement measures for the limited availability of RDTs

**Fig 5:**
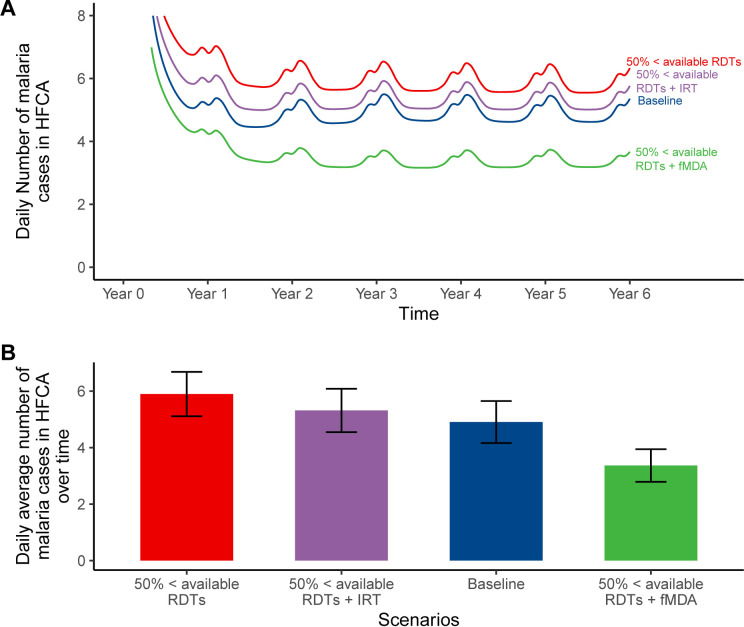
Impact predictions of RCD improvement measures for the limited availability of RDTs: (A) daily incidence in the HFCA; (B) average number of malaria cases over six years by scenario with their respective average uncertainty intervals. In this simulation, 50% of the secondary cases were tested, while fMDA (50% <available RDTs + fMDA) and a two-day reaction time (50% <available RDTs + IRT) were simulated as improvement measures. Baseline scenario had 20 CHWs, 140 radius, 3 days reaction time, no fMDA, and 0.84 RDT sensitivity.

Similar to the impact of a 50% shortage of CHWs, a 50% limited availability of RDTs (*red*) is better improved by incorporating fMDA (*green)* rather than improving the reaction time (*purple)* from three to two days (Fig 5). Incorporating fMDA (green) during an RDT shortage scenario reduces cases to approximately 123 annual cases per 1,000 population (3.37 daily cases per HFCA), compared to 215 annual cases per 1,000 population (5.90 daily cases per HFCA) when there is RDT shortage (red) as shown in Fig 5. This reduction results in fewer cases than the baseline (blue), which records 179 annual cases per 1,000 population (4.90 daily cases per HFCA). Conversely, improving reaction time (purple) during an RDT shortage reduces cases to approximately 194 annual cases per 1,000 population (5.31 daily cases per HFCA), which is higher than the baseline (Fig 5).

### Impact predictions of RCD improvement measures for sensitivity of RDTs

**Fig 6:**
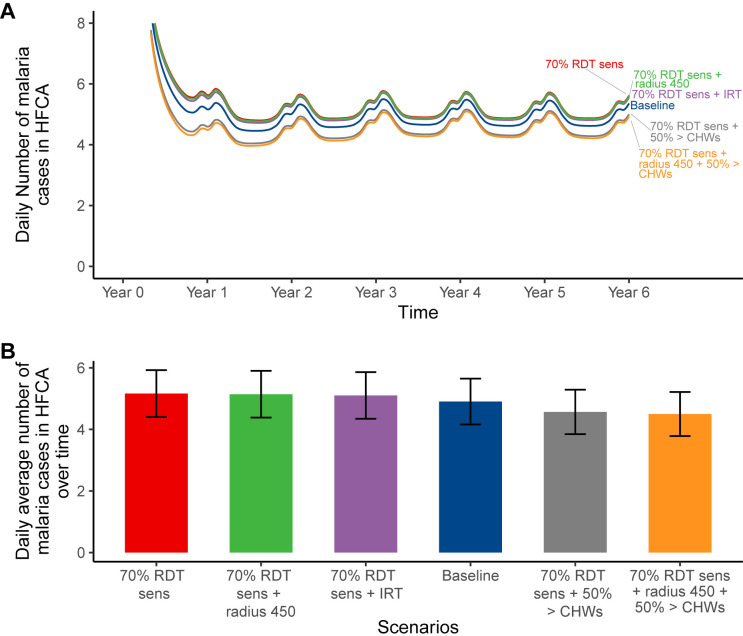
Impact predictions of RCD improvement measures for the limited sensitivity of RDTs: (A) daily incidence in the HFCA; (B) average number of malaria cases over six years by scenario with their respective average uncertainty intervals. In this simulation RDT sensitivity was reduced to 70% and increase of radius to 450 (70% RDT sens. + radius 450), increase of radius to 450m and CHWs by 50% (70% RDT sens. + radius 450 + 50%>CHWs), improved reaction time to two days (70% RDT sens. + IRT), and 50% more CHWs (70% RDT sens. + 50%> CHWs), as improvement measures. Furthermore, in (A), the blue, light blue and green share a similar trend, therefore obscuring each other, indicating negligible effect from the reduced RDT sensitivity. Baseline scenario had 20 CHWs, 140 radius, 3 days reaction time, no fMDA, and 0.84 RDT sensitivity.

The scenario of limited sensitivity of RDTs (*red)* which results in approximately 189 annual cases per 1,000 population (5.16 daily cases per HFCA) is best improved with an increase in the number of CHWs dedicated to RCD and an expanded radius (*orange*) as shown in Fig 6. Thus, combining “increased number of CHWs” by 50%, from 20 per HFCA to 30, and “expanding the radius to 450 meters” results in relatively fewer cases of approximately 164 annual cases per 1,000 population (4.50 daily cases per HFCA) even fewer than the baseline (*blue)* with 179 annual cases per 1,000 population (4.90 daily cases per HFCA). However, augmenting the CHW workforce alone also leads to an approximately similar trend (Fig 6). Notably, increasing the radius and reaction time independently had a negligible impact (Fig 6), suggesting that the increase in CHWs is the main contributor to case reduction in the increased CHWs and radius “combined” scenario.

### Impact predictions of RCD improvement measures for difficulties in finding residents in designated households

**Fig 7:**
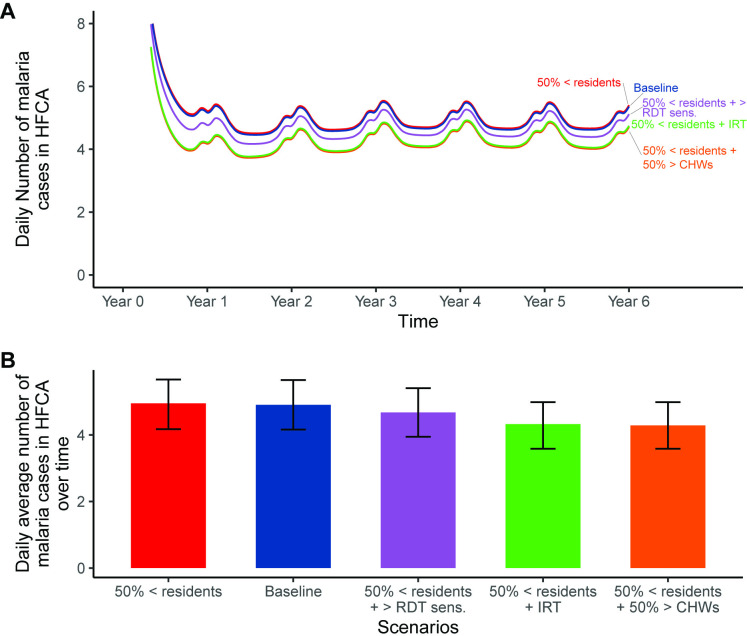
Impact predictions of improvement measures for difficulties in finding residents in designated households: (A) daily incidence in the HFCA; (B) average number of malaria cases over six years by scenario with their respective average uncertainty intervals. In this simulation, residents’ availability was reduced by 50% (50% <residents), while a two-day reaction time (50% <residents + IRT), 99% RDT sensitivity (50% <residents + RDT sens.), and 50% CHWs increase (50% <residents + 50> CHWs) were simulated separately as improvement measures. Baseline scenario had 20 CHWs, 140 radius, 3 days reaction time, no fMDA, and 0.84 RDT sensitivity.

Impact predictions of all investigated improvement measures for difficulties in finding residents in designated households resulted in relatively fewer cases than the baseline (blue) as depicted in Fig 7. The success of the improvement measures is attributed to the fact that the impact of not finding individuals in households (red almost sharing the same trend as blue baseline) had negligible effect on the overall number of cases (Fig 7). Thus, when only 50% of the residents were found resulted in approximately 181 annual cases per 1,000 population (4.95 daily cases per HFCA) compared to the baseline (purple) with 179 annual cases per 1,000 population (4.90 daily cases per HFCA). Among the simulated improvement measures, improving the reaction time (green) from three to two days and increasing the number of CHWs by 50% (orange), had the most impact at addressing the issue. As observed in Fig 7, improving the reaction time, or increasing the number of CHWs by 50% resulted in approximately 157 annual cases per 1,000 population (4.33 daily cases per HFCA), and 156 annual cases per 1,000 population (4.29 daily cases per HFCA), respectively. Conversely, increasing RDT sensitivity (purple), even up to 99%, had the least improvement but showed slightly lower infections of approximately 171 annual cases per 1,000 population (4.68 daily cases per HFCA) than the baseline (Fig 7).

## Discussion

We simulated an RCD focused model using parameters and data conforming to Zambia’s low transmission areas using a deterministic non-linear ordinary differential equation (ODE) model. Our primary objectives were to assess the impact of various literature-informed challenges affecting RCD to reduce malaria cases and their potential improvement measures. The analysis was undertaken with the purpose of informing the order for prioritizing the RCD challenges and guiding the appropriate improvement measures for each respective challenge. Considering that Zambia’s malaria risk stratification is done at a HFCA level and RCD is only conducted in low transmission HFCAs, the study was also set up at that level.

From the model validation and sensitivity analysis, we established that all parameters align with literature expectations regarding their relationship with malaria cases. Notably, the mosquito mortality rate had the highest impact on cumulative malaria cases. Statistically, the majority of non-intervention-related parameters were significantly associated with cumulative malaria cases. The insignificant relationship observed between the majority of intervention-related parameters and cumulative cases is likely due to the complex transmission dynamics of malaria, were factors such as CHWs, RDT sensitivity, reaction time, and time to seek treatment may not individually have a significant effect [30]. To account for seasonality, we used rainfall data to mimic Zambia’s transmission patterns because it coincides with malaria transmission trends [6]. As expected, this approach resulted in the model’s cases peaking between March and April, aligning with the usual peak malaria season in the country [3].

Scenario-wise, the simulated impact of RCD challenges on malaria cases within a HFCA revealed that a shortage of CHWs and RDTs had the most negative impact on RCD. In contrast, not finding individuals in households had the least impact. In scenarios where the availability of CHWs and RDTs was reduced by 50%, while keeping other parameters constant, annual malaria cases increased by approximately 22%. In both scenarios, the only effective countermeasure was the incorporation of fMDA, which resulted in an approximate 46% reduction in annual cases within the HFCA. However, using more sensitive RDTs and improving the response time to counter the 50% shortage in CHWs resulted in only four and 10 percent reductions in annual cases, respectively. Furthermore, in scenarios where RDT sensitivity was reduced to 70%, annual cases increased by five percent, while reducing the availability of individuals for testing by 50% led to approximately one percent increase in annual cases within the HFCA. In both scenarios, increasing the number of CHWs by 50% to offset their negative effects on RCD resulted in approximately 12% and 13% decreases in annual cases when using 70% sensitive RDTs and when availability of individuals was reduced by 50%, respectively. Additionally, in both scenarios, increasing CHWs by 50% as a countermeasure led to relatively fewer cases compared to adjusting the reaction time from three to two days or increasing the screening radius from an index case up to 450m from the initial 140m. Additionally, combining an increase in radius with a 50% increase in CHWs only reduced the number of annual cases by 13%, compared to the 12% reduction observed when only CHWs were increased to counter a 70% reduction in RDT sensitivity.

Considering that RCD operations in settings like Zambia are primarily conducted by CHWs, the number of index cases investigated directly depends on the number of CHWs, as set up in our model [4,5]. Our findings suggest that having more CHWs available for RCD results in more index case follow-ups. As such, our finding that the number of CHWs has the most substantial effect on the effectiveness of RCD aligns with Chitnis et al. (2019), who suggested that RCD is only successful in low transmission areas if many index cases are followed up [10]. However, Reiker et al. (2019) added that it is important to assess follow-up capacity rather than merely considering the actual number of cases. They argued that the potential number of index cases is limited by those who either do not seek official care or are asymptomatic, suggesting that the number of investigated index cases should be adjusted based on treatment-seeking behaviour [10,11]. On the other hand, increasing RDT sensitivity to address the shortage of CHWs and RDTs had minimal impact on incidence because positive cases linked to those not followed up—due to the CHW and RDT shortage—continue to transmit the infection, even if more secondary cases are detected using relatively sensitive RDTs from few index cases. Moreover, secondary cases missed by the less sensitive RDTs likely have low parasite densities, which limits their ability to contribute to further transmission [5]. This aligns with the findings of Chitnis et al. (2019), which highlight the critical importance of index case follow-up rates in determining the overall effectiveness of RCD.

Consistent with several studies, our research also indicates that MDA interventions may be the most effective alternative in various situations where RCD’s effectiveness is limited. For instance, Ntuku et al. (2022) noted that RCD requires notably more personnel time compared to fMDA and therefore uses fewer resources [31]. It is worth noting that integrating fMDA with RCD requires careful risk considering such as drug resistance, planning to address logistical challenges such as timely delivery of services and resource alignment, as well as ensuring the availability of supplies like antimalarials [31,32]. Baseline-wise, even though we modelled our baseline scenario with assumption that RCD was conducted perfectly, it did not result in zero infections in the HFCA over time. This supports findings from other studies that highlight that the ability of RCD to eliminate malaria depends on multiple factors, such as environmental risks and other archetypical factors, which our model may not have considered [5,9–11].

The study offers valuable insights into the challenges that impact the effectiveness of RCD and potential countermeasures. Implementers can utilize these insights to evaluate their resource capacity and combinations to suit an ideal RCD programme. For example, if the ratio of CHWs dedicated to RCD to the catchment population exceeds 1:1000 or if the health centre frequently experiences stockouts of RDTs, it might be necessary to consider fMDA as an alternative measure to interrupt infections in the HFCA. Otherwise, other improvements are likely to be ineffective. In situations where only less sensitive RDTs are available, the health facility may consider recruiting more CHWs to increase the number of index cases followed up as an improvement measure. However, failing to find secondary individuals should have relatively less priority compared to addressing other RCD challenges. In Zambia, the COVID-19 pandemic period provides a perfect example where the shortage of RDTs scenario is more applicable. In the same period, the Zambian healthcare system had 16,000 CHWs trained to undertake community-focused malaria interventions, but a number of them were inactive [3]. Reasons for the inactivity included the limited supply of antimalarial drugs (ACTs) and RDTs caused by COVID-19-induced supply chain interruptions [3]

It is worth noting that, the context governing the setup of how RCD is conducted may vary the outcomes of the results presented in this study. Furthermore, it is essential to note that this study faces certain limitations, including the ignoring of ecological feedback in mosquito dynamics introduced by the assumption that mosquitoes are at equilibrium with respect to changes in the human population. The assumption may be introducing bias because the mosquito populations change over time, which may influence the incidence differently. The assumption that CHWs possess perfect knowledge of how to conduct RCD is contrary to the evaluation by Searle et al. (2016). The evaluation established that the operational challenges for inadequate implementation of RCD may have also been due to various CHWs’ related inadequate technical capacity, such as the inability to distinguish the houses within the prescribed radius from the index case’s house [5,33]. Additionally, our model assumed that RCD was conducted daily, which is somewhat unrealistic given that CHWs have roles other than conducting RCD in the HFCA. Furthermore, the model used average values to estimate the number of index cases followed up by each CHW over the course of a year, resulting in an assumption of approximately three follow-ups per CHW annually. While this approach provides a general estimate, it may be overly restrictive for HFCAs with higher caseloads, such as those reporting cases higher than 60 per year, potentially underestimating the workload and impacting the accuracy of the model’s projections in such settings. Also, certain operational qualifiers/disqualifiers for RCD/MDA, such as travel history, season, and risk for drug resistance, were ignored. Similarly, the assumption that randomly testing within the population results in detections equivalent to if spatial locations were considered maybe causing negative bias in the “not finding individuals in households” simulation. Furthermore, the study assumed that antimalarial drugs were in abundant supply despite the shortage of RDTs, which may be unlikely, as alluded to earlier that both were in short supply during the peak of COVID-19 [3]. As such our model may be overstating the number of secondary cases that may be treated via MDA if RDTs are in short supply. Moreover, the model was not calibrated to any real data, hence the observed slight deviation in the number of annual cases beyond the low transmission threshold; therefore, all findings remain hypothetical and do not necessarily represent any true HFCA. Nevertheless, the outcome trends for the generated scenarios and the approach may be extended to other low-transmission catchment areas of Zambia with similar characteristics.

Based on results from [9,12–14,16,28,33–35] and this study, it is evident that the success of an RCD program in a Zambia-like setup highly depends on CHWs. However, their involvement is voluntary and primarily influenced by non-formal incentives, often provided by donors [33,36]. Finding sustainable means such as following World Health Organization’s 2018 guidelines for CHWs remuneration to ensure the sufficient availability of CHWs may guarantee continued RCD contributions towards maintaining stable malaria prevalence and ultimately contributing to achieving elimination [36]. The current CHWs’ contribution towards malaria programming and incentive situation warrants detailed research to optimize RCD implementation while considering potentially sustainable motivating incentives and monitoring and evaluation to maintain an optimal RCD programme. Furthermore, the coming of COVID-19, which interrupted several supply chain mechanisms including that for malaria supplies is a wake-up call to further look into improvement measures for other interventions other than RCD. The interventions of interest may include interruption of case management (antimalarial supplies and test kits), bednets, and IRS insecticides.

Lastly, considering the complexity of malaria transmission and several assumptions that were imposed on this study’s model. To make the modelling results more applicable across different contexts, we have research plans to stratify regions such as HFCAs into transmission archetypes based on malaria risk profiles, ecological, environmental, health system, and socioeconomic factors, and tailor interventions to suit specific archetypes, which may improve the outcomes and expedite the contribution to malaria elimination [37]. The archetyping approach may guide which low transmission areas will benefit most from RCD instead of alternative interventions or a combination of interventions. Conversely, interventions requiring fewer resources than RCD to avert the same number of malaria cases in certain archetypes may be opted for in place of RCD or a combination of interventions. Furthermore, the archetype-based targeting approach can also inform the optimization of interventions, including RCD. This way, the potential resources required to achieve elimination can be identified and planned for effectively. Outside our future research plans, the study warrants research to investigate CHW’s workload for RCD and feasibility studies to estimated resources that may be required to plan for the countermeasures highlighted in this study.

## Conclusion

This study contributes to the existing research literature by examining the impact of challenges faced by RCD on malaria cases at the HFCA level. It also highlights the effectiveness of potential improvement measures for these challenges. The study used mathematical modelling to simulate several scenarios to mimic RCD challenges and their respective potential improvement measures in a Zambia-like setting.

While challenges are inevitable during RCD implementation, our findings suggest that targeted and context-specific improvements can enhance or sustain its effectiveness in reducing malaria, thereby supporting elimination efforts. For example, increasing the number of CHWs by 50 mitigates the surge in cases, effectively offsetting the increase caused by using relatively less sensitive RDTs. However, the viability of these improvements is contingent upon addressing key resource constraints such as finding sustainable incentives for CHWs and identifying the underlying challenges before applying an improvement measure.

## Supporting information

S1 Text Force of Infection definition. (PDF)

S1 Fig Difference in cumulative cases after six years between the model with baseline (central) values and the models with lower and upper bounds for each non-intervention-related parameter. (PDF)

S2 Fig Difference in cumulative cases after six years between the model with baseline (central) values and the models with lower and upper bounds for each intervention-related parameter. (PDF)

S1 Table Statistical results highlighting the influence of both intervention and non-intervention parameters on malaria cases. (PDF)
