# Supplementary material for: Advancing malaria reactive case detection in a Zambia-like setting: A modeling study
Source: PLOS Glob Public Health. 2025 Feb 20;5(2):e0004288. doi: 10.1371/journal.pgph.0004288 (PMC11841873; doi:10.1371/journal.pgph.0004288)
Supplement: S2 Fig — (PDF) [file pgph.0004288.s003.pdf]

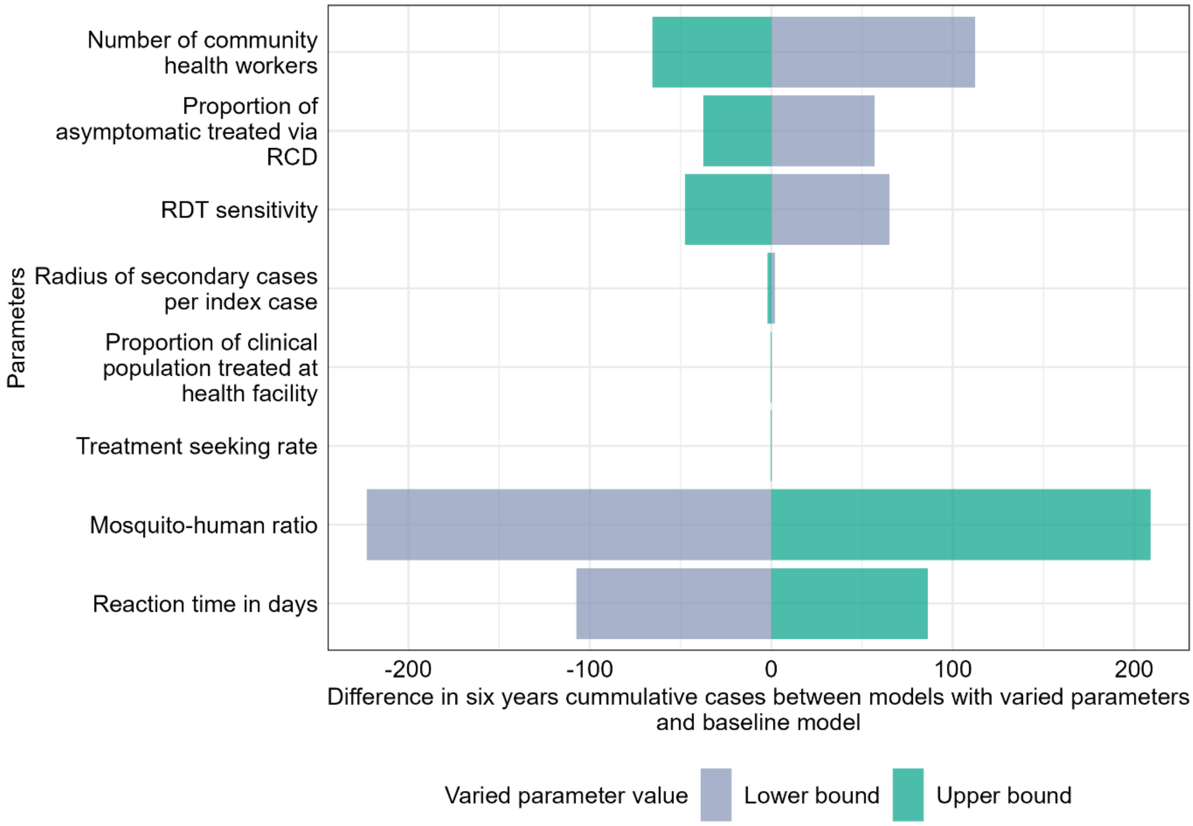

S2 Fig: Difference in cumulative cases after six years between the model with baseline (central) values and the models with lower and upper bounds for each intervention -related parameter.
