# Supplementary material for: Advancing malaria reactive case detection in a Zambia-like setting: A modeling study
Source: PLOS Glob Public Health. 2025 Feb 20;5(2):e0004288. doi: 10.1371/journal.pgph.0004288 (PMC11841873; doi:10.1371/journal.pgph.0004288)
Supplement: S1 Table — (PDF) [file pgph.0004288.s004.pdf]

**Sensitivity analysis regression results**

| term | estimate | std. error | statistic | p.value |
|---|---|---|---|---|
| (Intercept) | 2759.78 | 25720.97 | 0.11 | 0.91 |
| a | 38199.23 | 3007.91 | 12.70 | 0.00 |
| b | 99298.38 | 10279.36 | 9.66 | 0.00 |
| c | 30194.05 | 4977.93 | 6.07 | 0.00 |
| $\gamma_m$ | 58784.36 | 21371.74 | 2.75 | 0.01 |
| $\mu_m$ | -245408.57 | 27833.38 | -8.82 | 0.00 |
| $\gamma_h$ | -76912.81 | 137744.26 | -0.56 | 0.58 |
| τ | -31581.77 | 19713.80 | -1.60 | 0.11 |
| r | -13008.28 | 21979.40 | -0.59 | 0.55 |
| ρ | 6536907.15 | 268368.42 | 24.36 | 0.00 |
| m | 792.33 | 85.56 | 9.26 | 0.00 |
| pa | -70689.52 | 9266.46 | -7.63 | 0.00 |
| δ | 175448.38 | 177397.00 | 0.99 | 0.32 |
| da | -7953.90 | 15210.96 | -0.52 | 0.60 |
| ga | 2058.19 | 18610.55 | 0.11 | 0.91 |
| θ | 25514.53 | 5791.32 | 4.41 | 0.00 |
| ζ | 1848.35 | 10977.65 | 0.17 | 0.87 |
| CHW | -27.59 | 137.91 | -0.20 | 0.84 |
| radius | -4.62 | 8.18 | -0.56 | 0.57 |
| RDT sens. | -2032.48 | 10117.13 | -0.20 | 0.84 |
| Reaction time | 66.25 | 683.45 | 0.10 | 0.92 |
